# Role of miRNAs in CD4 T cell plasticity during inflammation and tolerance

**DOI:** 10.3389/fgene.2013.00008

**Published:** 2013-01-31

**Authors:** Apoorva Sethi, Neeraja Kulkarni, Sandip Sonar, Girdhari Lal

**Affiliations:** National Centre for Cell SciencePune, MH, India

**Keywords:** miRNA, T cell plasticity, tolerance, regulatory CD4 T cells, Th17 cells

## Abstract

Gene expression is tightly regulated in a tuneable, cell-specific and time-dependent manner. Recent advancement in epigenetics and non-coding RNA (ncRNA) revolutionized the concept of gene regulation. In order to regulate the transcription, ncRNA can promptly response to the extracellular signals as compared to transcription factors present in the cells. microRNAs (miRNAs) are ncRNA (~22 bp) encoded in the genome, and present as intergenic or oriented antisense to neighboring genes. The strategic location of miRNA in coding genes helps in the coupled regulation of its expression with host genes. miRNA together with complex machinery called RNA-induced silencing complex (RISC) interacts with target mRNA and degrade the mRNA or inhibits the translation. CD4 T cells play an important role in the generation and maintenance of inflammation and tolerance. Cytokines and chemokines present in the inflamed microenvironment controls the differentiation and function of various subsets of CD4 T cells [Th1, Th2, Th17, and regulatory CD4 T cells (Tregs)]. Recent studies suggest that miRNAs play an important role in the development and function of all subsets of CD4 T cells. In current review, we focused on how various miRNAs are regulated by cell's extrinsic and intrinsic signaling, and how miRNAs affect the transdifferentiation of subsets of CD4 T cell and controls their plasticity during inflammation and tolerance.

## Introduction

It has been speculated that about 1.5–3% of human genome encodes for the protein and remaining large fraction exist as non-protein coding sequences. Extra emphasis has been given on the effect of protein–protein and protein–DNA interaction on regulation of gene expression. Advancement in epigenetics and non-coding RNA (ncRNA) already proved that gene expression in a specific-cell can be fine-tuned based on the signals present in the tissue microenvironment (Esteller, 2011; Kaikkonen et al., 2011). New generation sequencing techniques such as RNA-seq, whole transcriptome analysis, and tiling arrays indicated that ~90% of genomic DNA is transcribed (Mortazavi et al., 2008; Pan et al., 2008; Wilhelm et al., 2008; Lindberg and Lundeberg, 2010). ncRNA like ribosomal RNA, tRNA, small nucleolar RNA (snoRNA), and small nuclear RNA (snRNA) are also transcribed from the genome but never translate into protein. These ncRNA controls the transcription and translation of the several genes (Costa, 2010). ncRNAs are comprises of a class of heterogeneous member that ranges in size from few to several hundred nucleotides. Based on their size and origin, they are identified as microRNAs (miRNAs), PIWI-interacting RNAs (piRNAs), long non-coding RNAs (lncRNAs), recently identified enhancer RNAs (eRNAs), promoter-associated RNAs (PARs), transcription start site-associated RNAs (TSSa-RNAs), and transcription initiation RNAs (tiRNAs) (Taft et al., 2009; Esteller, 2011). ncRNAs perform several important functions that control the development, survival, cell migration, cell differentiation, apoptosis, immune response, maintaining genome stability, and stress responses by regulating the gene expression (Kaikkonen et al., 2011; Tomankova et al., 2011). Among the various ncRNAs, miRNAs are extensively studied in the context of their role in dynamic regulation and micromangement of transcriptome in the various cells.

Several subsets of CD4 T cells have been identified, and they all are developed in thymus from a common precursor T cells. Based on the cytokine secretion and function, these cells are classified as Th1, Th2, Th9, Th17 and regulatory CD4 T cell (Treg). Recent studies showed that these cells are not terminally differentiated but have potential to differentiate into other CD4 T subset (Lal and Bromberg, 2009; O'Shea and Paul, 2010; Lal et al., 2011; Gao et al., 2012). The differentiation of different subsets of CD4 T cells are regulated by cytokine and other extracellular signals present in the tissue microenvironment (Lal and Bromberg, 2009; O'Shea and Paul, 2010; Lal et al., 2011; Gao et al., 2012). Under the influence of different extracellular inflammatory stimuli, Tregs which have potential to suppress inflammation and help in the maintenance of tolerance can transdifferentiate into the pathogenic Th1 and Th17 cells (Xu et al., 2007; Lal et al., 2011; Kanno et al., 2012). It has been reported that under inflammatory condition CD4^+^Foxp3^+^ Treg (nTreg) down-regulates Foxp3 expression (a master transcription factor for nTreg) and differentiated into the CD4 follicular helper T cells (Tfh) (Tsuji et al., 2009). It has also been reported that loss of Foxp3 in nTreg-induced IL-4 expression and converted into Th2 lineage leading to development of autoimmune colitis (Wang et al., 2010). It has been shown that helminth antigens induced the differentiation of Th2 cells into Tfh cells (Zaretsky et al., 2009). Tfh cell can also differentiate into other effector subsets such as Th1, Th2, and Th17 cells (Lu et al., 2011). Bending et al. reported that under lymphopenic condition, Th17 cells differentiated into Th1 cells (Bending et al., 2009). Similarly, in presence of IFN-γ Th2 cells re-programmed into GATA3^+^T-bet^+^ cell subset and showed combined function of Th2 and Th1 cells (Hegazy et al., 2010). All these studies clearly demonstrated that CD4 T cells possess fair amount of plasticity to differentiate into various subsets of CD4 T cell lineage, and inflammation in the tissue microenvironment play an important role in this cellular re-programming. The detail cellular and molecular mechanisms that regulate plasticity of CD4 T cell differentiation and function are not completely understood.

Recent studies have suggested strong association of miRNAs in many inflammatory and autoimmune diseases (Chong et al., 2008; Tomankova et al., 2011; Contreras and Rao, 2012). In this review, we have discussed recent advances in the understanding of miRNA biogenesis and its role in the development, differentiation, and function of different subset of CD4^+^ T cells, and how miRNAs regulate the plasticity of CD4 T cells during inflammation and tolerance.

## miRNA biogenesis

**Figure 1:**
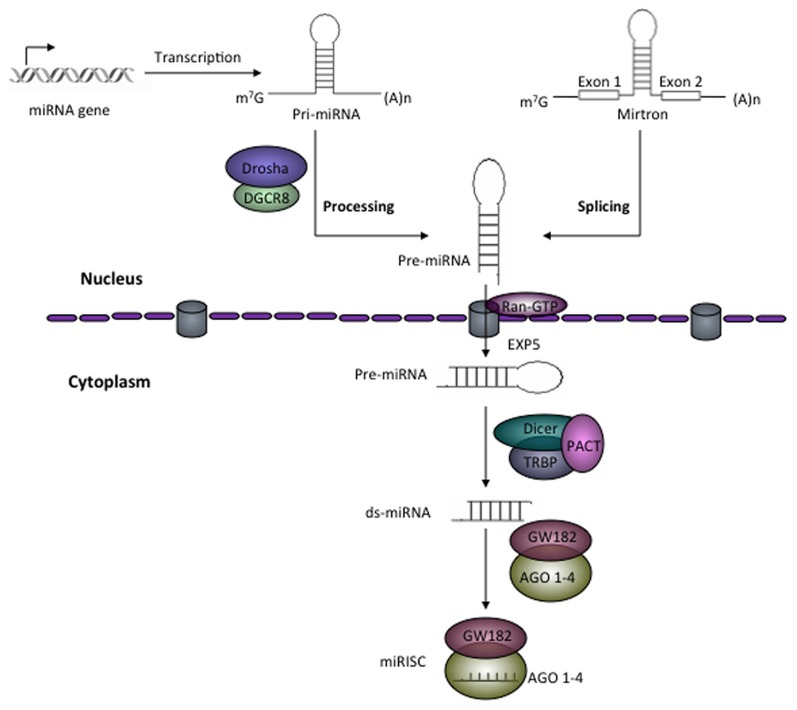
**Schematic representation of miRNA biogenesis.** RNA polymerase II transcribes the miRNA gene and produces hairpin structured pri-miRNA that contains 5′ cap and 3′ poly-A tail. In the nucleus, Drosha in association with DGCR8, a ds-RNA-binding protein generates about 55–70 nt long precursor miRNA (pre-miRNA). In eukaryotes, some pre-miRNAs are generated from mirtrons as a product of splicing reaction. With the help of Ran-GTP bound exportin-5 pre-miRNAs are exported from the nucleus to cytoplasm. In the cytoplasm, Dicer together with TAR RNA-binding protein (TRBP) or PACT removes the loop and generates miRNA duplex from pre-miRNAs. The miRNA duplex (ds-miRNA) is then loaded into Argonaute (AGO) proteins (in the case of humans AGO 1–4). One of the strands of ds-mRNA called guide strand retained in the AGO and with other effector protein factors forms miRNA-induced silencing complex (miRISC).

Majority of miRNAs from introns of protein coding host genes are transcribed by RNA polymerase II (RNA pol II). Some of miRNAs are also formed by splicing of RNA polymerase III products. Biogenesis of miRNA is a highly regulated multi-step process, initially carried out into the nucleus but later processed matured in the cytoplasm (Figure 1). In the nucleus, primary miRNA transcripts (pri-miRNA) a double-stranded-RNA containing stem-loop are produced as a result of RNA pol II driven transcription. Pri-miRNAs are further processed by Drosha (RNase III family member) and a cofactor DGCR8, a double-stranded-RNA-binding protein which recognize about 10 bp near hairpin structure and cleaves both strands of stem at sites near the base of primary stem-loop, and resulting hairpin precursor miRNA (pre-miRNA) (Lee et al., 2003). Drosha-independent processing of the miRNAs is also reported, especially for those miRNA derived from intronic region as a product of splicing reaction (Berezikov et al., 2007; Ruby et al., 2007). Later, pre-miRNAs transported to the cytoplasm by exportin-5 (Figure 1). Exportin-5 needs another cofactor Ran bound to GTP for export function, and this Ran-GTP bound exportin-5 recognizes 3′ overhangs of pre-miRNA (Bohnsack et al., 2004; Lund et al., 2004). In the cytoplasm, loop region of pre-miRNA is removed by another RNase-III family member known as “Dicer.” Dicer is associated with TAR RNA-binding protein (TRBP) or PACT and produce about 22 nt long miRNA duplex from pre-miRNA (Hutvagner et al., 2001; Chendrimada et al., 2005; Lee et al., 2006). Together, Dicer, TRBP, or PACT along with Argonaute 1–4 (AGO 1–4) form a complex known as RISC-loading complex (RLC). RLC loads the guide strand (one of the two strands which has unstable base pairing at its 5′ end) into the RNA-inducing silencing complex (RISC) and process the duplex-miRNA (Chendrimada et al., 2005). In human, AGO proteins bind to the guide miRNA strand, interacts with glycine-tryptophan protein (GW182) and finally form the major component of miRISC (Jakymiw et al., 2005; Liu et al., 2005). miRISC regulates the gene expression either by suppressing translation or by mediating the deadenylation and further degradation of target mRNA (Figure 1).

## Regulation of miRNA biogenesis

It has been reported that miRNA biogenesis is a very tightly regulated process, and a number of regulatory mechanisms with negative feedback control participate in the generation of pri-miRNA (Johnston et al., 2005; Kim et al., 2007; Turner and Slack, 2009). Post-transcription processing of pri-miRNA provides another layer of regulatory mechanism for miRNA biogenesis such as Drosha inhibitory molecules like lin-28 inhibits processing of let-7 primary transcript (Newman et al., 2008). It has been reported that mutation in TARBP2 protein, an integral component of Dicer1-containing complex, leads to destabilization of processing machinery. It is also found that TARBP2 protein controls post-transcriptional processing of miRNA and promotes tumor growth (Melo et al., 2009). It has been reported that DGCR8 required for the Drosha-mediated miRNA processing and negatively regulates generation of pre-miRNA (Triboulet et al., 2009). Nuclear export of pre-miRNA by Ran-GTP bound exportin-5 can also functionally regulate the generation of functional miRNA (Lee et al., 2008). RNA editing has been reported to regulate the miRNA generation by changing the sequence of pri-miRNA or pre-miRNA leading to decreased (Kawahara et al., 2007a) or increased (Kawahara et al., 2008) efficiency of miRNA generation, and also alteration of miRNAs target specificity (Kawahara et al., 2007b).

## Mechanisms of miRNA function

**Figure 2:**
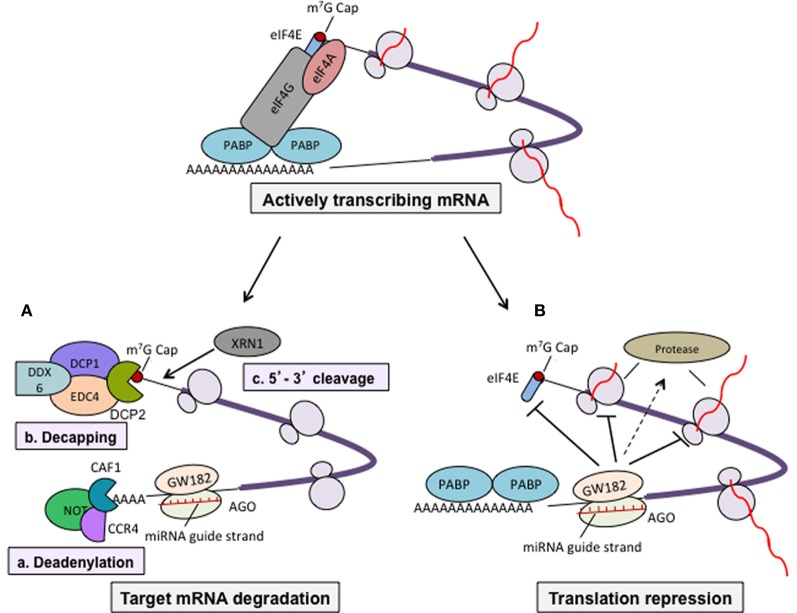
**Mechanisms of miRNA-mediated gene regulation.** miRNA-mediated gene regulation mostly occurred by two ways; affecting mRNA stability in the cells or suppressing translation of target mRNA. miRNA-induced silencing complex (miRISC) consisting of miRNA guide strand, AGO 1–4 (in humans) and among others trinucleotide-repeat-containing protein GW182, altogether perform regulating function. Actively transcribing mRNA forms a circular structure via the interaction of eIF4F (eIF4A, eIF4E, eIf4G) with 5′ cap and poly-A-binding protein (PABP) that increases the efficiency of translation. **(A)** Regulation of mRNA stability. Binding of miRISC to 3′-UTR of target mRNA recruit the deadenylase complex consisting of CAF1-CCR4-NOT that removes the 3′ poly-A tail. It has also been reported that deadenylation may also leads to removal of cap by DCP (DCP1 and DCP2)-mediated decapping (Behm-Ansmant et al., 2006). DCP1 and DCP2 along with some other factors such as EDC4, DDX6 removes 5′ m^7^G cap and decapped mRNA provides a platform for 5′–3′ exonuclease (XRN1 is one of characterized members) leading mRNA degradation (Parker and Song, 2004). **(B)** Regulation of target mRNA translation. miRNAs can also regulate translation by suppressing translation initiation (reports suggest that GW182 compete with eIF4E for 5′ cap binding during initiation stage), elongation, co-translational protein degradation, and premature termination.

It is well-established that miRNAs regulate gene expression post-transcriptionally, mostly by either repressing the translation or affecting the stability of target mRNA in the cytoplasm (Figure 2). Most of the miRNAs function in the cytoplasm but some miRNAs are also reported to be localized in the nucleus (such as miR-29b) (Hwang et al., 2007) and some are secreted outside of the cells (Valadi et al., 2007). miRNAs were also reported to positively regulate the gene expression (Vasudevan and Steitz, 2007; Vasudevan et al., 2007; Orom et al., 2008). This suggests that various miRNAs can function differently in different cell-types and can control the development and differentiation of cells.

### miRNAs regulate gene expression by degrading target mRNA

There is a reciprocal correlation between expression of miRNAs and their target mRNAs (Huntzinger and Izaurralde, 2011). This suggests that miRNAs function by targeting mRNA degradation. miRNA binds to the target mRNA with partial base pair complementarity. Studies in animal cells and *in vitro* cell-free extract have revealed that binding of miRISC to its target mRNA recruits deadenylase complex consist of CAF1/CCR4/NOT which removes 3′ poly-A tail from target mRNA (Behm-Ansmant et al., 2006; Wu et al., 2006). The deadenylation process followed by decapping mediated by DCP1 and DCP2 together with other protein factors. Decapped mRNAs are targeted by XRN1 a 5′–3′ exonuclease present in the cytoplasm leading to degradation of mRNA (Parker and Song, 2004; Behm-Ansmant et al., 2006). Therefore, removal of both the ends of mature mRNA by miRNA-mediated mechanism affects the stability of mRNA in the cytoplasm and renders them to undergo degradation (Figure 2).

### miRNAs regulate gene expression by repressing translation of target mRNA

Several studies using wide variety of cells as well as in cell-free system suggested that miRNAs repress the translation of target mRNA at both initiation and post-initiation stages. There are several evidences that support the inhibition of translation at initiation stage such as target mRNAs having functional m^7^Gppp-cap at their 5′ end were repressed whereas a synthetic non-functional 5′Appp-cap and internal ribosome entry site (IRES)-mediated translation was unaffected (Humphreys et al., 2005; Mathonnet et al., 2007). Using bi-cistronic construct, Pillai et al. showed that only first cistron which had 5′ cap was affected but cistron having IRES did not show any change in translation (Pillai et al., 2005). Similarly, it has been reported that AGO proteins compete with eIF4E for binding at 5′ cap of target mRNA and inhibit the translation (Kiriakidou et al., 2007). These evidences clearly suggest that miRNA-mediated repression at translation initiation stage is cap-dependent. In contrast, it has also been reported that miRNAs repress mRNAs that are translated by IRES-dependent mechanism (Petersen et al., 2006; Lytle et al., 2007). Other mechanisms regulated by miRNA (acting at post-initiation of translation) include inhibition of elongation steps, degradation of protein during ongoing translation, and premature termination of translation are reviewed nicely elsewhere (Huntzinger and Izaurralde, 2011).

## Role of miRNAs in the immune system

**Figure 3:**
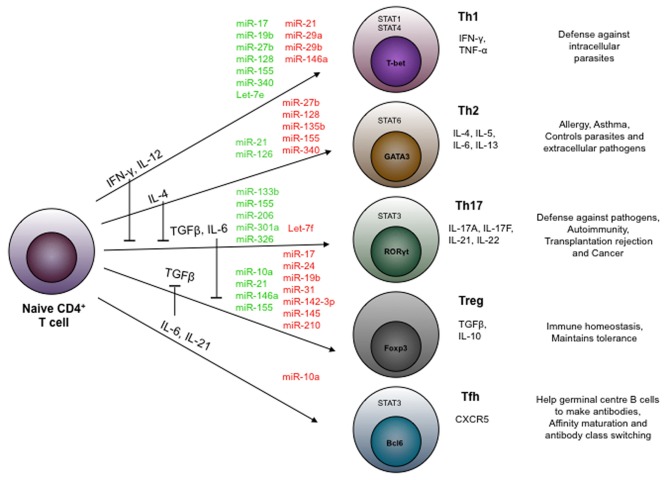
**miRNAs control the differentiation of CD4^+^ T helper cell subsets.** miRNAs regulates the differentiation of different effector (Th1, Th2, Th17, and Tfh) and regulatory (Treg) sub-population of CD4^+^ T helper cells. miRNAs shown in green color are reported to positively regulate whereas those in red color negatively regulate their differentiation.

miRNAs are known to control many important processes such as development, survival, proliferation, differentiation, and function of immune cells. Several miRNAs have been reported to control the expression of cytokines, chemokines, growth factors, cell adhesion molecules, co-stimulatory molecules, and transcription factors (Table 1). For example, over-expression of miR-181 in hematopoietic precursor cells can direct the lymphoid differentiation into B cell lineage and inhibit development of T cell (Chen et al., 2004). Deletion of Dicer is embryonic lethal. However, conditional deficiency of Dicer only in early developmental stage of T cells (CD4^−^CD8^−^ double-negative thymocytes; using lck-Cre model) (Lee et al., 2001) or later T cell developmental stage (CD4^+^ single-positive thymocytes; using CD4-Cre model) reported to have no change in the mature CD4 or CD8 lineage choices (Cobb et al., 2005; Muljo et al., 2005). However, deletion of Dicer in T cells showed generation of reduced numbers of total TCR α/β thymocytes in thymus (Cobb et al., 2005; Muljo et al., 2005). This suggests that miRNA plays an important role in the development of T cells in thymus. It has been reported that deficiency of Dicer in CD4 T cells (CD4-cre model) did not blocked IFN-γ production (Th1 lineage cytokine) even under Th2 polarization condition. It suggests that miRNA expression was required in each specific stage in order to control the function of the specific lineage of CD4 T cells (Muljo et al., 2005). miRNAs were also reported to mediate antibody class switching, formation of germinal center and activation of antigen presenting cells (APCs), and secretion of pro-inflammatory cytokines (Baltimore et al., 2008). CD4^+^ T cells occupy central position in the adaptive immune system, and they provide help to B cells and CD8^+^ T cells for their differentiation and effector function. Importance of various miRNAs in the differentiation and function of different subset of CD4 T cells are depicted in Figure 3. Role of miRNAs in the immune responses are well-reviewed elsewhere (O'Connell et al., 2012). In present review, we are focusing on role of miRNAs in the plasticity and its function in different subset of CD4^+^ T cells during inflammation and tolerance.

**Table 1:** **miRNA in CD4 T cells**.

**Sr. No.**	**miRNAs**	**Targets**	**Function and profile of miRNA**	**Cells/animal models**	**References**
1	miR-155	SOCS-1		CD4^+^ Th cells	Stahl et al., 2009
2	miR146	AP1 (Transcription factor for IL-2)		Jurkat T cells	Curtale et al., 2010
3	miR-340	3′UTR of IL-4 mRNA	Expression of miR-340 increased in memory T cells of MS patients	Memory T cell of MS patients	Guerau-de-Arellano et al., 2011
4	miR-155	Inhibits c-MAF expression		CD4^+^ T cells	Rodriguez et al., 2007
5	miR-26	3′UTR of IL-6 mRNA		Human lung epithelial A549 cell line	Jones et al., 2009
6	miR-206 and miR-133b	ETS-1	Expression of miR-206 and -133b regulated by IL-23 signaling	CD4^+^ T cell and CCR6^+^ γδ T cells	Haas et al., 2011
7	miR-326	ETS-1	Up-regulated in patients with Multiple Sclerosis (MS)	CD4^+^ TH17 cells from patients with MS	Du et al., 2009
8	miR-155		Up-regulated in EAE model	CD4^+^ TH17 cells from EAE model	O'Connell et al., 2010
9	miR-301a	PIAS3	miR-301 over-expressed in EAE and supports Th17 development by targeting IL 6/STAT3 pathway	Myelin-specific CD4^+^ cells in EAE	Mycko et al., 2012
10	miR-29	T-bet and Eomes	Down-regulates production of IFN-gamma by targeting T-bet and Eomes	CD4^+^ T cell	Ma et al., 2011
11	miR-155	Inhibits IFNγRa signaling		Activated CD4^+^ T cell	Banerjee et al., 2010
12	miR-19b	Down-regulates PTEN		CD4^+^ T cell	Jiang et al., 2011
13	miR-17, miR-18a, and miR-20a	CXCR5 mRNA	Expression of all the three miRNA is down-regulated by bcl-6	Tfh cells	Yu et al., 2009
14	miR-21 and miR-148a	DNMT1	Up-regulated the expression of CD70 by demethylating its promoter	SLE	Pan et al., 2010
15	miR-155	CTLA-4	Enhances T cell proliferation by suppressing CTLA4	Atopic dermatitis	Sonkoly et al., 2010
16	miR-29	T-bet and Eomes	Down-regulated IFNγ expression by down-regulating T-bet and Eomes	CD4^+^ T cell	Steiner et al., 2011
17	miR-128 and miR 27b	BMI-1	Expression of miR-128 and miR-27b increased in Naive CD4^+^ T cells in patients with MS	Naive CD4^+^ T cell in Multiple Sclerosis	Guerau-de-Arellano et al., 2011
18	miR-326	3′UTR of ETS-1	Expression of miR-326 up-regulated in MS and EAE	TH17 cells in EAE and MS	Du et al., 2009
19	miR-21			nTreg cells	Rouas et al., 2009
20	miR-31	3′UTR of Foxp3	miR-31 under expressed in Treg cells	nTreg cells	Rouas et al., 2009
21	miR-10a	3′UTR of BCL-6	miR-10a highly expressed in nTreg cells, and its expression was induced by TGF-β and retinoic acid	T helper cells	Takahashi et al., 2012
22	Let-7e	3′UTR of IL-10 and IL-13	Over-expressed during EAE and promotes development of Th1 and Th17 cells	CD4^+^Th1 cells in EAE model	Guan et al., 2013
23	miR-126	3′-UTR of DNMT-1	Indirectly inhibits PU.1 and act as a negative regulator of GATA3	Mouse model of Allergic asthma, CD4 T cells in SLE	Mattes et al., 2009; Zhao et al., 2011
24	miR-24	3′UTR of CTLA4 and 3′UTR of Foxp3	Expression of miR-24 down-regulated in CD4^+^CD25^+^ CD127^low^ Tregs	Human peripheral blood CD4^+^CD25^+^ CD127^*low*^ Tregs	Fayyad-Kazan et al., 2012
25	miR-145	3′UTR of CTLA4 and 3′UTR of Foxp3	Expression of miR-145 down-regulated in CD4^+^CD25^+^ CD127^low^ Treg	Human peripheral blood CD4^+^CD25^+^ CD127^low^ Tregs	Fayyad-Kazan et al., 2012
26	miR-210	3′UTR of CTLA4 and 3′UTR of Foxp3	Expression of miR-210 down-regulated in CD4^+^CD25^+^ CD127^low^ Tregs	Human peripheral blood CD4^+^CD25^+^ CD127^low^ Tregs	Fayyad-Kazan et al., 2012

## Role of miRNA in regulation of Th1/Th2 balance

Helper CD4^+^ T cells function both in contact-independent (by secreting cytokines and chemokines) and contact-dependent manner (by interacting with co-stimulatory molecules). IFN-γ secreted by Th1 cells activates the APCs (macrophages, dendritic cells, and B cells), and induces increased surface expression of class-II MHC and co-stimulatory molecules on APCs, and boosts the antigen presenting function of APCs. APCs secrete IL-12 which promotes phosphorylation of STAT4 that in turn induces expression of transcription factor T-bet in CD4 T cells and control the production of IFNγ. IL-12-induced IFN-γ initiates a positive feedback loop which further promotes production of IL-12. Positive feedback-induced production of IL-12 gives a strong Th1 response.

Several miRNAs are known to regulate the secretion of IFN-γ and IL-12 cytokines which required for the differentiation of Th1 cells. NK cells play important role in the innate immunity. It has been shown that miR-132, miR-200, miR-212a regulate IL-12 production in NK cells (Huang et al., 2011). These miRNAs negatively regulate STAT4 and inhibit expression of IL-12 and IFN-γ (Huang et al., 2011). In the helper CD4 T cells, miR-29a and miR-29b inhibit production of IFN-γ by targeting T-bet and Eomes, lineage-specific transcription factors that regulate the differentiation of Th1 cells (Ma et al., 2011; Steiner et al., 2011). Th1/Th2 cells are known to be involved in the various autoimmune and inflammatory diseases. Increased expression of miR-27b, miR-128, and miR-340 in CD4^+^ T cells has been reported in multiple sclerosis (Guerau-de-Arellano et al., 2011). These miRNAs promote effector function of Th1 by inhibiting the proliferation of Th2 cells. miR-128 represses the expression of Bim1, a proapoptotic molecule required for stabilization of GATA3 (transcription factor for Th2 cell lineage), whereas miR-340 down-regulates the expression of IL-4 and together these miRNAs direct the differentiation of Th1 cells (Guerau-de-Arellano et al., 2011). Increased expression of miR-155 is reported in experimental autoimmune encephalomyelitis (EAE) and promotes differentiation of inflammatory Th17 cells (O'Connell et al., 2010). miR-155 also suppresses c-Maf a transactivator of IL-4 and inhibits proliferation of Th2 cell (Rodriguez et al., 2007). Deficiency of miR-155 leads to excessive Th2 response and decreased IL-17 and IL-22 production in collagen-induced arthritis (Bluml et al., 2011). Over-expression of miR-155 in activated T cells leads to the differentiation of Th1 response (Banerjee et al., 2010). miR-155 target site is also found on 3′-untranslated region of IFN-γRα, and inhibition of miR-155 leads to induction of IFN-γRα (Banerjee et al., 2010). It has been reported that miR-146 expression into naïve CD4 T cells was low but showed increased expression into effector Th1 (Monticelli et al., 2005) and Treg (Cobb et al., 2006), but not in Th2 cells (Monticelli et al., 2005). miR-150 expressed in naïve CD4 T cells but down-regulated after activation and differentiation into Th1, Th2, and Treg (Monticelli et al., 2005; Cobb et al., 2006; Curtale et al., 2010). These results suggest that various miRNAs involve in the regulation of Th1 and Th2 cell differentiation and function.

It has been reported that miR-17-92 cluster was involved in the generation of Th1 cells. Deficiency of miR-17-92 leads to the reduced expression of T-bet and IFN-γ and promotes differentiation of the Foxp3^+^ Treg (Jiang et al., 2011). Analysis of functional targets showed that miR-17 targets TGFβRII and cAMP-responsive element-binding protein 1 (CREB1) and both of these molecules are known to involved in Treg differentiation. Therefore, this cluster displays a unique mechanism that reciprocally regulates Th1 and Treg generation. Wu et al. showed that miR-21 expression was inversely correlated with IL-12 production in murine APCs which further leads to reduction in IL-12-dependent differentiation of Th1 cells (Wu et al., 2012).

miR-29b targets T-bet and IFN-γ which are signatures of Th1 cells (Smith et al., 2012). Furthermore, IFN-γ in CD4 T cells were found to enhance miR-29b expression via STAT1 binding to the promoter of miR-29ab1, and forms a negative feedback loop (Smith et al., 2012). Recently, it has been reported that expression of miRNA Let-7e was up-regulated in an encephalitogenic CD4 T cells in EAE mice. Silencing of Let-7e expression *in vivo* using antagonist anti-miR increased Th2 response and inhibited Th1 and Th17 responses leading to attenuation of EAE (Guan et al., 2013). This suggests that Let-7e has important role in balancing pro-inflammatory and anti-inflammatory responses by reciprocally tuning the function of subset of CD4 T cells. Foster's group has showed that miR-126 level was correlated with the generation of inflammatory Th2 cells in the mouse model of allergic asthma. Suppression of miR-126 expression in the airway resulted into down-regulation of IL-4, IL-5, and IL-13 production by Th2 cells and up-regulation of Oct binding factor 1 (OBF.1), which is a negative regulator of transcription factor PU.1 (Brunner et al., 2007). PU.1 inhibits the expression of Th2-specific transcription factor GATA3. Thus, this study provided a mechanism by which miR-126 promotes the generation of Th2 cells.

All these evidences indicate that several miRNAs regulates the differentiation and function of Th1 and Th2 and controls the pathology.

## Role of miRNA in treg cells

Regulatory CD4^+^CD25^+^ T cells (Tregs) play an important role in maintaining the homeostasis and immunological tolerance by suppressing pathogenic CD4^+^ T cell response (Lal and Bromberg, 2009; Lal et al., 2009). It has been reported that deletion of Dicer in the late stage of T cell development did not interfere the process of development and differentiation of T cells (Cobb et al., 2005; Muljo et al., 2005). However, lck-Cre-mediated conditional deletion of Dicer into CD4 T cells reduces the number of Foxp3^+^ nTreg in thymus and secondary lymphoid organ (Cobb et al., 2006). The lack of Dicer in the CD4^+^ single positive stage blocks the TGFβ-induced differentiation of Foxp3^+^ Treg *in vitro* (Cobb et al., 2006). Deletion of Dicer in Foxp3^+^ Treg (Foxp3-GFP-hCre model) leads to loss of suppression function and induces their differentiation into T helper memory phenotype (Zhou et al., 2008). Dicer deficiency in Foxp3^+^ cells leads to the development of fatal autoimmune diseases resembling the Foxp3 knockout phenotype (Zhou et al., 2008). These observations clearly suggest that miRNAs at different stages of T cell differentiation regulate different functions. Dicer is not only involved in the processing of miRNA but also involved in the processing of short hairpin RNA (shRNA). Therefore, phenotype reported in Dicer-deficient animals accounts for the loss of not only miRNA but also shRNA. RNase III enzyme Drosha is specifically required for generating miRNA and deletion of Drosha in the CD4 T cells leads to spontaneous T cell activation, inflammatory diseases, and premature lethality (Chong et al., 2008). Ablation of Drosha specifically in the Foxp3^+^ T cells leads to early onset of lymphoproliferative disease as observed in T cell or Foxp3-deficient mice (Chong et al., 2008). These reports suggest that different miRNA processing enzymes in CD4 T cells can affect the differentiation and function of nTreg.

miR-155 is highly expressed in the Treg (Zheng et al., 2007). It has been shown that Foxp3 binds to the promoter region of *bic* gene, which is transcribed into miR-155 precursor mRNA (Marson et al., 2007). Deficiency of miR-155 showed reduced Treg in the thymus and periphery, however Treg from miR-155 deficient animal do not show any defect in suppressive function both *in vitro* and *in vivo* (Kohlhaas et al., 2009). Furthermore, Rudensky's laboratory showed that deficiency of miR-155 leads to the increased expression of suppressor of cytokine signaling 1 (SOCS1) leading to impaired activation of signal transducer and activator of transcription factor 5 (STAT5) (Lu et al., 2009). STAT5 signaling is required for the survival and function of Tregs. Rudensky's group also reported that miR146a is also prevalently expressed in Treg cells and critical for Treg function. In addition, they also showed that deficiency of miR146a increases the number of Tregs but impaired their function. miR146a directly targets STAT1 which is a key transcription factor required for IFN-γ signaling and Th1 differentiation thus helps in Treg function (Lu et al., 2010). miR146a knocked out mice showed increased Th1 phenotype which is similar to specific depletion of SOCS1 (a negative regulator of STAT1) in Foxp3^+^ Treg leading to breakdown of tolerance and massive autoimmune inflammation (Lu et al., 2010).

CD4 T cell activation requires low levels of intracellular cAMP. It has been reported that cAMP play an important role in contact-dependent manner to control the suppressive function of nTreg. nTreg contains high level of cAMP compared to the naïve or effector CD4 T cells (Bopp et al., 2007). nTreg releases intracellular cAMP into effector T cells through tight junction and suppresses the effector cell function (Bopp et al., 2007). It has been shown that activated helper CD4 T cell contains high levels of phosphodiesterase, an enzyme responsible for turnover of cAMP. This enzyme is down regulated in the nTreg (Marson et al., 2007; Zheng et al., 2007). It has been reported that blocking of cAMP degradation by phosphodiesterase 4 inhibitor lead to increased Tregs suppressive function (Bopp et al., 2009). Elevated levels of intracellular cAMP blocks IL-12 signaling pathway in target cells and thus suppress its differentiation into Th1 lineage. In the same context, Huang et al. showed that down-regulation of miR142-3p is essential for Treg function (Huang et al., 2009). miR-142-3p maintains low levels of intracellular cAMP by targeting adenylyl cyclase (AC) 9 mRNA (Huang et al., 2009). In Treg cells, Foxp3 directly down-regulates miR-142-3p expression and keeps the AC9/cAMP pathway active and elevates intracellular cAMP levels (Huang et al., 2009). Thus, down-regulation of miR142-3p is required for suppressor function of Treg cells.

Comparison of miRNA expression between human naïve CD4 T cells with Treg showed altered expression of five signature miRNAs (miR-21, -31, -125a, -181c, and -374) in Treg (Rouas et al., 2009). miR-31 and miR-125a were strongly down-regulated while miR-21, -181c, and -374 were found to be significantly up-regulated in Treg cells. miR-31 is known to target Foxp3 mRNA and negatively regulates its expression. miR-21 might be involved in positive regulation of Foxp3 expression. Functions of remaining three miRNA in Tregs are needs to be explored (Rouas et al., 2009). miRNA analysis of Tregs from normal and diabetic patients showed significant increased expression of miR-510 and decreased expression of miR-342 and miR-191 (Hezova et al., 2010). Hezova et al. hypothesized that down-regulation of miR-342 in Tregs and impairement of Treg function in type 1 diabetic patients may be associated with disease progression. For better understanding the functional significance of differential expression of miR-510, miR-342, and miR-191 in Treg during type 1 diabetes need to be further investigated.

It has been shown that Foxp3 expression in thymic-derived Treg (nTreg) are more stable compared to the induced Treg (iTreg) (Zhou et al., 2008; Lal and Bromberg, 2009). It has been shown that nTreg possess demethylated CpG residues in their regulatory regions of the *Foxp3* gene whereas it was completely methylated in the iTreg (Floess et al., 2007; Lal et al., 2009). Exposure of iTreg to DNA methyltransferase inhibitors leads to increased stability in the Foxp3 expression (Baron et al., 2007; Lal et al., 2009). Inflammatory signals modulated the epigenetic marks and affect the *Foxp3* expression in Tregs (Li et al., 2010; Lal et al., 2011). Recently, it has been shown that nTreg expresses high levels of miR-10a and provides stability of Foxp3 expression whereas iTreg induced with TGF-β alone does not express miR-10a, leading to decreased stability of Foxp3 expression. It has been reported that addition of retinoic acid (RA) together with TGF-β induces miR-10a expression in iTreg (Jeker et al., 2012). O'Shea's group showed that in TGF-β and RA iTreg, miR-10a targets transcriptional repressor Bcl6 and co-repressor Ncor2, and inhibits the differentiation of iTreg to Tfh cells (Takahashi et al., 2012). It has been shown that miR-10a inhibits T-bet-dependent Th17 differentiation in iTreg and reduces the severity of EAE (Takahashi et al., 2012). Recently, it has been demonstrated that miR-24 and miR-210 target Foxp3 mRNA and regulates human Treg differentiation (Fayyad-Kazan et al., 2012).

These studies clearly suggest that a number of miRNAs not only regulates the development of Treg but also controls the plasticity and function of Treg. Disruption in Treg migration, differentiation, and function is known to have direct impact on controlling the inflammation and tolerance.

## Role of miRNA in Th17 cells

Naive CD4^+^ T cells under the influence of IL-6 and TGF-β differentiate into distinct subset called Th17 which secretes IL-17A, IL-17F, and IL-22. Th17 cells are reported to play an important role in clearance of pathogens, graft rejection, and autoimmune diseases (Segal, 2010; Wilke et al., 2011). miR-326 has been shown to be up-regulated in Th17 cells (Du et al., 2009). Over-expression of miR-326 in mice-induced severe EAE which was marked by increased number of Th17 cells in CNS as well as increased expression of Th17 signature cytokines (IL-17A, IL-21, IL-22, IL-23R) in Th17 cells (Du et al., 2009). E26 transformation-specific-1 (Ets-1), a known negative regulator of Th17 (Moisan et al., 2007) and acts as a functional target of miR-326 that represses the translation of Ets-1 mRNA (Du et al., 2009).

miR-155 has been reported to be linked with the development of inflammatory CD4 T cells. miR-155^−/−^ mice have been found to produce defective Th1 and Th17 cells during EAE as well as in mouse model of delayed type hypersensitivity (O'Connell et al., 2010). Naive CD4^+^ cells from miR-155^−/−^ mice showed defects in *in vitro* differentiation of Th17 cells. GM-CSF-derived activated myeloid dendritic cells from miR-155^−/−^ mice were reported to produced IL-6, IL-23p19, IL-23p40, and TNF-α in significantly reduced amount. Since, IL-6 and IL-23 secreted by dendritic cells are essential for Th17 cell development. It has been suggested that miR-155 expression in dendritic cells is required for development of inflammatory Th17 cells (O'Connell et al., 2010). miR-155 is also associated with several autoimmune diseases such as EAE (Murugaiyan et al., 2011), arthritis (Kurowska-Stolarska et al., 2011), systemic lupus erythematosus (SLE) (Leng et al., 2011), chronic gastritis, and colitis (Oertli et al., 2011). In contrast to these reports, expression of miR-155 has been found to negatively regulate Th17 cells in acute coronary syndrome patients (Yao et al., 2011). Another miRNA, Let-7f was found to control the expression of IL-23R in memory CD4^+^ T cells. Memory CD4 T cells showed decreased Let 7f and up-regulation of IL-23R in MS (Li et al., 2010). It has been established that IL-23/IL-23R signaling is essential for the maintenance of Th17. Using EAE model, Mycko et al. showed that miR-301a helps in promoting Th17 cell differentiation (Mycko et al., 2012). They also reported that silencing of miR-301a in CD4 T cells resulted in down-regulation of Th17-specific gene expression and did not affect the *Tbx21 and Foxp3* expression. Further investigation in different subsets of T helper cells suggested that miR-301a-mediated STAT3 phosphorylation is essential for the Th17 cell differentiation program. Along with this effect, miR-301a is known to inhibit PIAS3, a molecule known to interfere with STAT3 signaling pathway (Chung et al., 1997). Altogether, these studies suggested that expression of miR-301a in CD4 T cells contribute to the generation of inflammatory Th17 cells. IL-17 secreting murine Th17 and γδ T cells has been found to have increased expression of miR-133b and miR-206. These miRNAs were reported to present in syntenic to the *Il17a/f* locus and their expression was co-regulated with IL-17 production (Haas et al., 2011). Further investigation in human primary CD4 T cells revealed that only CD4^+^IL-17A^+^IFNγ^+^ Th17 cells but not CD4^+^IL-17A^−^IFNγ^+^ Th1 cells express both miR-133b and miR-206. These reports suggest that various miRNAs differentially regulate the transdifferentiation of Th17 cells. In brief, there are several miRNAs that can modulate Th17 function and affect the pathogenesis of various diseases. Modulation of Th17 function and its epigenetic plasticity under inflammatory and tolerogenic conditions need further thorough investigation. How do miRNAs fine-tune the Th17 function in secondary lymphoid organs and inflamed microenvironment is not yet clear and need active investigation.

## Future perspective

During infection or inflammatory conditions set for the Th1 differentiation such as infection of *Toxoplasma gondii* or *Mycobacterium tuberculosis*, Tregs acquires Th1 phenotypes (Koch et al., 2009; Oldenhove et al., 2009). Further analysis suggested that T-bet^+^ Treg controls the Th1 response whereas T-bet^−^ Treg promotes Th1/Th17-mediated autoimmune diseases (Koch et al., 2009; O'Connor et al., 2010). Neutralization of IFN-γ in graft-vs.-host disease model ablates alloreactive Treg function leading to necrosis and graft rejection (Sawitzki et al., 2005). A transcription factor IRF4 was known to regulate the differentiation of CD4 T cell subsets (Lohoff et al., 2002). Conditional deletion of IRF4 in the Treg leads to the increased Th2 response and pathogenesis of the diseases (Zheng et al., 2009). Apart from the transcription factors, now several investigators are trying to understand how do miRNAs regulate the differentiation and function of the cells. Transgenic and knockout mouse models provide a valuable tool to understand the physiological significance of each of these miRNAs and understanding the function and its targets in the immune cells may revolutionize the field.

Like protein coding RNA, a numbers of ncRNAs are also induced by inflammatory signals. The timing of miRNA expression, cell-types, and mode of action may have very unique role in the cell differentiation and physiological function. The interesting part of ncRNA-mediated gene regulation is that they are processed quickly and do not have to undergo translation process to become functionally active. This makes them to response promptly to the inflammatory signals and can fine-tune the transcription. miRNAs are involved in the various tissue inflammations and control both immune cells (which initiate antigen-specific response and produces pro-inflammatory cytokines) and tissue stromal cells (that magnify the response by producing chemokines that recruits monocytes and granulocytes). For example, microglia cells in the brain down-regulate miR-124 leading to its activation during EAE (Ponomarev et al., 2011) whereas miR-155 and miR-326 expression increased in Th17 cells, together increase the inflammation and pathogenesis of the diseases (Du et al., 2009; O'Connell et al., 2010). There are several targets that are controlled by miRNA (Table 1) which suggests that a therapeutic approach can be employed by targeting a specific or multiple miRNAs simultaneously in an effort to achieve a strong and potent effect on a tissue-specific inflammation. Antigen-specific Tregs provide a valuable cellular therapy to induce transplantation tolerance and control the autoimmunity. However, understanding the plasticity of antigen-specific Treg and its differentiation into antigen-specific effector T cells *in vivo* due to exposure to inflammatory signals in the body imposes a caution using such cellular therapy. Future studies to understand the function of various miRNA in the Th1, Th2, Th17, and Treg and its affect on inflammatory signals may provide a novel therapeutic strategy to manipulate the antigen-specific Treg for clinical use.

## Funding

This work was supported by Department of Biotechnology, Government of India (grant reference number BT/RLF/Re-entry/41/2010, BT/03/IYBA/2010, and BT/PR4610/MED/30/720/2012). Neeraja Kulkarni and Sandip Sonar are the junior research fellow of Council of Scientific and Industrial Research, Government of India.

### Conflict of interest statement

The authors declare that the research was conducted in the absence of any commercial or financial relationships that could be construed as a potential conflict of interest.

## Abbreviations

AGO: Argonaute
APCs: Antigen presenting cells
EAE: Experimental autoimmune encephalomyelitis
miRNA: microRNA
MS: Multiple sclerosis
ncRNA: Non-coding RNA
RISC: RNA-induced silencing complex
RITS: RNA-induced transcriptional silencing
shRNA: Short hairpin RNA
Tregs: Regulatory CD4 T cells.
